# Supplementary material for: Physiological and clinical effects of low-intensity blood-flow restricted resistance exercise compared to standard rehabilitation in adults with knee osteoarthritis–Protocol for a randomized controlled trial
Source: PLoS One. 2023 Dec 14;18(12):e0295666. doi: 10.1371/journal.pone.0295666 (PMC10721028; doi:10.1371/journal.pone.0295666)
Supplement: Attachment — Submitted filename: Response to Reviewers_16.11.23.docx [file pone.0295666.s003.docx]

**Replies to Review Comments on Manuscript PONE-D-23-14776**

We like to thank the Editor and Reviewers for their constructive comments and suggestions, which have helped to improve the quality of the manuscript. Below we have addressed all comments raised by the reviewers in a point-to-point fashion, including a listing of the specific modifications performed in the revised manuscript. Page and line numbers refer to the revised manuscript, unless otherwise stated. All changes have been indicated by red texting in the Revised Manuscript with Track Changes.

**Journal requirements**

(1) Please ensure that your manuscript meets PLOS ONE's style requirements, including those for file naming.

Author’s reply:

The revised manuscript has been thoroughly checked and now meets PLOS ONE’s requirements for style requirements.

(2) Your ethics statement should only appear in the Methods section of your manuscript. If your ethics statement is written in any section besides the Methods, please delete it from any other section.

Author’s reply:

The Ethics and dissemination statement has now been moved to the Materials and Methods section (p. 18-19, lines 455-480).

(3) Please review your reference list to ensure that it is complete and correct. If you have cited papers that have been retracted, please include the rationale for doing so in the manuscript text, or remove these references and replace them with relevant current references. Any changes to the reference list should be mentioned in the rebuttal letter that accompanies your revised manuscript. If you need to cite a retracted article, indicate the article’s retracted status in the References list and also include a citation and full reference for the retraction notice.

Author’s reply:

The reference list has been thoroughly reviewed as suggested (p. 23-34, lines 571-886).

**Editor Suggestions**

(1) Maybe add a hypothesis, following the aim?

Author’s reply:

Thanks for this constructive suggestion. A hypothesis has been added after the Aim (p. 2, lines 39-41 and p. 5, lines 121-123). “*We hypothesize that 12 weeks of BFR will lead to superior improvements in pain, muscle mass and mechanical muscle function compared to SR.*”

(2) Consider if TIDieR could support the description and transparency of the intervention even further?

Author’s reply:

The TiDieR checklist has been completed and is submitted along with the revised manuscript.

**Editor Comments**

(1) Please revise the manuscript gramatical and check spelling.

Author’s reply:

The revised manuscript has been revised for grammar and spelling.

(2) Line 123 it is stated that assesment will take place after 24 weeks. Line 260 assesment is reported to take place after 36 weeks, and also in line 445? ClinincalTrials says 6 months (24 weeks).

Author’s reply:

Thank you for pointing out this inconsistency. Sentences have been rephrased accordingly and it is now stated that follow-up questionnaires will be assessed 24 weeks after the intervention period throughout the entire manuscript (p. 6, lines 133-134 and p. 11, lines 270-271: *“…completed 24 weeks after the intervention period.”*).

P. 16, line 445 in the original manuscript seems to have been a mistake. The sentence has been rephrased to *“The raw data will be stored for five years after completion of the trial with restricted access to the data”* (p. 19, lines 486-487 in the revised manuscript).

(3) Line 263, what standardized test procedures, please report, refer or provide reference for the standardized descriptions.

Author’s reply:

The sentence has been rephrased to clarify the test procedures in question (p. 11, lines 272-273): *“…**the assessor will be thoroughly trained in performing the tests according to the specific procedures involved in each test modality (elaborated below).”*

(4) Line 327, maybe in bold instead of Italic?

Author’s reply:

We see Editor’s point. The sentence has been changed to bold instead of italic (p. 15, line 340): *“****Secondary outcomes related to mechanical muscle function”***

(5) Regarding the power calculation - please clarify or consider why you calculate the required n, based on within-group differences, when you describe the primary outcome as the between-group difference? It´s a superiority trial is it not?

Author’s reply:

We thank Editor for the question and see the inconsistency in the sentence phrase. This is a superiority trial, and the power calculation is based on between-group differences. The sentence has been rephrased accordingly (p. 20, lines 494-499): *“The estimated number of participants in the study are based on the primary outcome variable, KOOS-Pain subscale (58), with the assumption that a between-group change of 10 points is considered clinically relevant (58, 60) assuming a standard deviation (SD) of 15 points (11, 60). To achieve a statistical power of 80 % with a significance level of 0.05 and an expected between-group change of 10-points* *following 12 weeks training (primary endpoint) using a two-sample pooled t test required 37 participants in each group”*

(6) The 10 point difference you report as considered clinical relevant is that within-group or between-group. PLease clarify.

Author’s reply:

Thank you for pointing out this inconsistency. Sentence has been rephrased to clarify (p. 20, line 495): *“…with the assumption that a between-group change of 10 points…”*

**Reviewer #1**

(1) Line 135: If block randomization will be used, state the block size.

Author’s reply:

Thank you for your question. We are not using block randomization, but as stated a stratified randomization (p. 6, lines 145-149).

(2) Line 455: Indicate the statistical testing method which achieves 80% power.

Author’s reply:

Thank you for pointing out this missing information. It is now included in the sample size section (p. 20, lines 497-499): *“To achieve a statistical power of 80 % with a significance level of 0.05 and an expected between-group change of 10-points following 12 weeks training (primary endpoint) using a two-sample pooled t test required 37 participants in each group”*

(3) Line 464: Indicate the underlying covariance structure that will be used in the mixed linear model or the criteria for selecting it.

Author’s reply:

Thank you for pointing out this inconsistency. Sentence has been rephrased and it is now stated which covariance structure will be used (p. 20, lines 505-506): *”…mixed linear model analysis with an autoregressive covariance structure will be used…”*

(4) Line 471: Clarify if the mixed model or some other statistical method will be used to correlate the primary outcome to the secondary outcomes.

Author’s reply:

Thank you for pointing out this missing information. It has now been included in the statistical analysis section (p. 20, lines 514-516): *“The primary outcome will be correlated to the various secondary outcomes using multiple linear regression analyses.”*

(5) The abstract indicates that one-way analysis of variance (ANOVA) will be used for data analysis; however, ANOVA is not mentioned in the “Statistical analysis” section. Clarify.

Author’s reply:

Thank you for pointing out this inconsistency. It is now included in the statistical analysis section (p. 20, lines 511-514): *“All outcome parameters (primary, secondary) will be analyzed using a one-way analysis of variance model to analyze between-group mean changes. The model includes changes from baseline to 8- and 12-weeks. Pre-to-post intervention comparisons will be analyzed using a mixed linear model approach”*

(6) State the software that will be used for the statistical analysis.

Author’s reply:

Thank you for pointing out this missing information. It is now stated which statistical software that will be used (p. 20, lines 517-518): *“All statistical analyses will be performed in a blinded fashion SPSS (Version 22, IBM SPSS Statistics).”*

**Reviewer #2**

(1) The topic is not focused. The topic should be neither be too short nor too long. In your case it is too lengthy.

Author’s reply:

We see and understand reviewer’s point. We have chosen to be very thorough in describing both the topic and the overall project as it is a protocol article.

(2) It is not necessary to write the type of research (i.e. a randomized controlled trial) b/c it has its own section (in the method and material part).

Author’s reply:

We understand reviewer’s point. However, we have chosen to be clear in the lengthened title that this is a protocol for a randomized controlled trial.

(3) The abstract is not written in an intelligible fashion and in Standard English. For example it says the aim is to examine the effect of. You better to use was. Because this manuscript is completed, it is not a proposal. The other thing is that almost all the manuscript was written in future tense. You used the future ‘’will be…’’ several times throughout the manuscript. Thus, you should write in Standard English. Because you already submitted a completed manuscript, so why you write in the form of future tense.
    Author’s reply:

We understand reviewer’s point. We would use past tense in a manuscript with a completed study. However, we are still collecting data and therefore writing in future tense.

(4) I didn’t see the RESULT and CONCLUSION of the study in the abstract part, so why?

Author’s reply:

We understand reviewer’s question. This is a manuscript for a protocol article why we have no data to publish, and therefore cannot conclude anything yet.

(5) Table-3 Page-12 under the outcome variables, you write as Table-3, however; I didn’t find this table-3 in your manuscript. So why?

Author’s reply:

Thank you for pointing out this inconsistency and missing table. There is no Table 3, but Table 2 is missing. The sentence has been rephrased and Table 2 has been included in the manuscript (p. 13, lines 282-283).

**Other changes made in the revised manuscript**

P. 1, line 10 and lines 26-27: An asterisk and e-mail address have been added to corresponding author.

P. 1, lines 10-111 and 22-24: Two of the co-authors have new affiliations, which have been added to the Author Affiliations section.
